# ADNP Upregulation Promotes Bladder Cancer Cell Proliferation *via* the AKT Pathway

**DOI:** 10.3389/fonc.2020.491129

**Published:** 2020-11-09

**Authors:** Shuai Zhu, Zhenzhou Xu, Yong Zeng, Ying Long, Gang Fan, Qi Ding, Yuheng Wen, Jian Cao, Tao Dai, Weiqing Han, Yu Xie

**Affiliations:** ^1^ Department of Urology, The Affiliated Cancer Hospital of Xiangya School of Medicine, Central South University, Hunan Cancer Hospital, Changsha, China; ^2^ Clinical Translational Research Center, The Affiliated Cancer Hospital of Xiangya School of Medicine, Central South University, Hunan Cancer Hospital, Changsha, China; ^3^ Department of Urology, Huazhong University of Science and Technology Union Shenzhen Hospital, The 6th Affliated Hospital of Shenzhen University Health Science Center, Shenzhen, China

**Keywords:** activity-dependent neuroprotective protein, cell cycle, proliferation, bladder cancer, AKT pathway

## Abstract

**Background:**

Activity-dependent neuroprotective protein (ADNP), which is involved in embryonic development and neurogenesis, has been proven to be upregulated in some human tumors. However, its role in bladder cancer (BC) has never been studied.

**Objective:**

We aimed to investigate the mechanisms by which ADNP promotes the progression of BC.

**Methods:**

ADNP expressions in BC cell lines and paired BC and adjacent normal tissues were measured by quantitative real-time PCR (qRT-PCR), Western blot, and immunohistochemistry. Colony formation, Cell Counting Kit-8 (CCK-8), trypan blue exclusion assay, flow cytometry, and nude mice tumorigenesis assay were performed to explore the effects of ADNP on growth of BC *in vivo* and *in vitro*. The impacts of ADNP on AKT signaling pathways were measured by Western blot.

**Results:**

The expression of ADNP mRNA and protein was significantly upregulated in BC tissues compared with adjacent normal tissues. Immunohistochemical analysis of 221 BC and 51 adjacent normal tissue paraffin sections indicated that ADNP expression was significantly associated with histological classification and pathological T and N stages. Survival analysis revealed that patients with high ADNP expression have worse prognosis with respect to overall survival and progression-free disease. ADNP knockdown markedly delayed propagation of BC *in vitro* and the development of BC *in vivo.* ADNP overexpression showed the opposite effect. In addition, ADNP can markedly promote G1-S cell cycle transition in BC cells. On the molecular level, we confirmed that ADNP mediated acceleration of G1-S transition was associated with activation of the AKT pathways in BC.

**Conclusion:**

ADNP is overexpressed in BC and promotes BC growth partly through AKT pathways. ADNP is crucial in predicting the outcome of BC patients and may be a potential therapeutic target in BC.

## Introduction

Bladder cancer (BC), particularly urothelial carcinoma, is one of the most prevalent urinary system malignancies (1). Despite significant improvements in surgical and chemotherapeutic treatment, incident cases and recurrence rates continue to increase by 5% (2). Approximately 70% of all incident cases are non-muscle-invasive bladder cancer (NMBC), while 30% are muscle-invasive bladder cancer (MIBC) (3). NMBC rarely progresses but recurs easily, leading to high treatment costs because of the need for long-term monitoring (4). Meanwhile, MIBC, to some extent, is more aggressive and prone to metastasis, and thus the 5-year survival rate is only 50% (5). This dismal prognosis highlights the urgent need for accurate prognostic markers for BC that can be used for both treatment planning and follow-up.

Activity-dependent neuroprotective protein (ADNP), which locates in the chromosome 20q12–13.2 and encodes a protein with nine zinc fingers and a bipartite nuclear localization signal (6), has been found to protect neural activity against external stimuli in glial cells (7). As a transcription factor, ADNP is extremely conserved and highly expressed in antenatal life. It interacts with some components of the SWI/SNF chromatin remodeling complex such as BAF250a, BRG1, and BAF170 and mediates genes involved in brain formation and neurodevelopment (8, 9). The ADNP-deficient mouse model showed an inability to close neural tube and died at the embryonic stage (9). Previous studies have also identified that ADNP functions as a responsive genetic factor of vasoactive intestinal peptide and has a critical role in axonal transport, dendritic spine plasticity, and autophagy (10, 11). Importantly, ADNP is closely associated with various clinical illnesses, such as epilepsy, intellectual disabilities, and autism spectrum disorders (12).

The role of ADNP in tumorigenesis has recently gained profound research attention, and ADNP is closely linked with tumor growth, particularly in various cancers, including breast, ovarian, pancreatic, and colon cancer (13). According to the previous research, ADNP loss-of-function mutations have been found to be related to carcinogenesis (14). It was reported that ADNP can suppress cellular migration, invasion, and proliferation *via* the WNT signaling pathway in colon carcinoma (15) and triple-negative breast cancer (16). By contrast, the activation of the ADNP signaling system, mediated by an endogenous pituitary adenylate cyclase-activating polypeptide, can increase the resistance of malignant peripheral nerve sheath tumor to H_2_O_2_-induced death with serum starvation (17). It suggests that ADNP may also act as an oncogene in certain cellular contexts. Pascual et al. reported that ADNP overexpression could induce activation of the AKT pathway (18), which plays a major role in cancer cell proliferation and cell cycle development (19). Moreover, p53 protein, regulated by ADNP/SWI/SNF complex, is inactivated in cancer (20), leading to unlimited cell growth (21). Owing to this dual characteristic, the mechanisms of ADNP in BC are poorly understood. Our previous study showed that ADNP was significantly upregulated in BC. Therefore, we hypothesize that ADNP can stimulate the proliferation of BC cells *via* AKT pathway.

In this study, we investigate the role of ADNP in BC growth and identify the underlying mechanism whether ADNP can regulate the proliferation and cell cycle in BC cells *via* activating AKT signaling pathway. By activating AKT signaling pathway, ADNP enhance the proliferation of BC cell *in vitro* and *in vivo*. These findings establish evidence that ADNP have an important role in BC tumorigenesis.

## Materials and Methods

### Cell Lines and Tissue

We analyzed 221 paraffin-fixed specimens of human bladder urothelial carcinoma tissues as well as 51 neighboring conventional bladder transitional cell tissues collected between January 2005 and December 2007 at the Affiliated Cancer Hospital of Xiangya School of Medicine, Central South University. In addition, 20 cases of BC fresh surgical specimens were collected from patients hospitalized in our hospital from September to December in 2017. The adjacent normal control was collected at least 2 cm away from the tumor margin (22). The collected cancer tissue and its adjacent normal tissue have been identified under the microscope. All patients with BC were diagnosed by histopathology and treated with radical cystectomy or tumor resection. Human BC cell lines (BIU87, T24, 5637, and TCCSUP) were acquired from the Institute of Cancer Prevention and Treatment, Sun Yat-Sen University (Guangzhou, China) and human normal urothelial cells (SV-HUC-1) were acquired from Department of life science, Hunan Normal University (Changsha, China). BC cell lines were cultivated in RPMI-1640 media (Gibco, USA) supplemented with 10% fetal bovine serum (Gibco, USA), 1% streptomycin, and penicillin, and the SV-HUC-1 cells were cultivated in F-12k media (Gibco, USA) supplemented with 10% fetal bovine serum (Gibco, USA), 1% streptomycin, and penicillin. All the cells were cultured under 5% atm CO_2_ at 37°C.

### Quantitative Real-Time PCR (qRT-PCR)

RNA extraction was performed using TRIzol reagent (Invitrogen, USA) according to the manufacturer’s instructions. RNA purity was assessed *via* spectrophotometry (A260/A280 = 1.8–2.0). M-MLV transcriptase (BioRAD, USA) was used to generate cDNAs according to the manufacturer’s instructions. Quantitative real-time polymerase chain reaction (RT-PCR) was performed using 1 µg cDNA, 0.4 µl primer pairs for the interest gene, and 5 µl 2X SYBR green (BioRAD, USA) *via* LightCycler480 RT-PCR System (BioRAD, USA) under these amplification conditions: one cycle of 95° for 30 s, followed by 35 cycles in 95° for 15 s, 95° in 10 s, 65° in 60 s, and a final cycle of 97° for 1s. The comparative threshold cycle method (2^-ΔΔCT^) was applied for estimating the relative gene expression among BC tissues and corresponding normal bladder urothelial tissues. Triplicate PCR amplifications were performed for each sample. The primer sequences for ADNP amplification were as follows: forward: 5′-CATCCTGCGTCTGGACCTGG-3′; reverse: 5′-TAATGTCACGCACGATTTCC-3′.

### Western Blot Analysis

The cells and tissues were washed with phosphate-buffered saline (PBS) and then lysed using RIPA buffer (0.5% sodium deoxycholate, 0.1% SDS, 50 mM Tris, and 150 mM NaCl, pH 8.0) with protease inhibitor mixture (Roche, USA) and phosphatase inhibitors (Roche, USA) at freezing condition for 15 min. The protein levels were measured with BCA Protein Assay Reagent kit (Thermo Scientific, USA). A 10% SDS-polyacrylamide gel was used to separate tissue lysate aliquots containing 20 μg protein. These were subsequently moved to PVDF membranes (Millipore), and the membranes were consequently blocked for 2 hours with TBST buffer with 5% skim milk at 22°C, and incubated at 4°C with primary antibodies overnight. We then added peroxidase-conjugated secondary antibodies and performed ECL (Cell Signaling Technology, 12757) visualization. Band enumeration was conducted using densitometric analysis software (Bio-Rad). GAPDH expression was used as the internal standard to standardize expression of the supplementary proteins. The primary antibodies were as follows: anti-ADNP (1:1000, Proteintech, USA), anti-GAPDH, CDK4, CDK6, Cyclin D1, Cyclin B1, p-cdc-2, p-Rb, E2F1, p53, MDM2, AKT, p-AKT, and p21 (1:1000, Cell Signaling Technology, USA). The secondary antibodies were HRP-Goat-anti-Rabbit Ig G and HRP-Goat-anti-Mouse Ig G (1:1000, Cell Signaling Technology, USA).

### Immunostaining 

The paraffin-fixed tissues were cut into 5 µm sections and heated for 2 h at 60°C. Slices were deparaffinized using xylene, rehydrated in graded alcohols, and then treated with 0.3% hydrogen peroxide in methanol. Subsequently, they were immersed in antigenic recovery buffer of EDTA and heated in a microwave for antigen retrieval. Thereafter, the slices were blocked using 1% BSA and anti-ADNP (1:200, Abcam, ab199120) and then incubated for 12 h at 4°C. After washing in PBS, we added anti-rabbit secondary antibody (Zymed, USA) onto the tissue slices and then incubated them with peroxidase-tagged streptavidin (Zymed, USA). Thereafter, the slices were submerged in 3.3′-diaminobenzidine and subsequently counterstained, dried, and fixed using hematoxylin.

Two independent experienced observers blinded to the histopathological diagnosis and clinical information reviewed and rated the extent of formalin- and paraffin-fixed immunostaining. They had been exposed to professional training in immunohistochemical staining scoring. Scores were based on combination of the ratio of completely stained cancer cells to the staining power. Tumor cell ratio was graded as 0 for no positive tumor cells; 1, < 10%; 2, 10%–25%; 3, 26–75%; and 4, > 75% positive tumor cells. Meanwhile, the extent of staining was rated as 1 for negative staining; 2, weak staining (light yellow); 3, adequate staining (yellow brown); and 4, heavy staining (brown). The staining index was calculated by multiplying the fraction of positive tumor cells by the staining power score. The staining index (total 0, 1, 2, 3, 4, 6, 8, 9, and 12) was used to estimate ADNP expression in bladder tissues, with scores ≥ 6 and < 6 considered as high and low expression, respectively. Five areas at a magnification of 400× were chosen randomly, and each area was scored separately according to the staining index (23). The score of a paraffin slide was obtained by the average score of these five areas.

### Cell Transfection

The plasmids overexpressing ADNP (ViGene, Shandong, China) and vector (ViGene, Shandong, China) were used to target the ADNP gene. Through subcloning whole cDNA human ADNP to a pMSCV-retro-puro vector (Clontech, Palo Alto, CA), an ADNP construct was expressed. For the ADNP knockdown, three ADNP-shRNA (KD-1: CAACATGACTGATGGAGTA; KD-2: GCAAATGCCTCTACTGTAA; KD-3: TAGTAAGACTGCTGACAAA) and a non-silencing shRNA (NC) were purchased from GeneChem, Shanghai, China. Cells were transfected with shRNA when they reached 30%–50% confluence. The cells were then incubated for 72 h in a 1 μg/mL puromycin (Sigma-Aldrich) medium for selection before transfection. After selection, cell replicas were confirmed *via* western blot analysis. The fabricated stable cell lines were preserved in 0.1 μg/mL puromycin medium.

### Cell Proliferation Assay

Approximately 2,000 stably transfected cells were distributed onto 96-well plates. Once attached, they were prepared as per their individual study procedure. Subsequently, 20 µl Cell Counting Kit−8 reagent (CK04, Dojindo Kumamoto, Japan) was supplemented to individual wells and retained for 2 h. Vmax microplate spectrophotometer (Molecular Devices, Sunnyvale, CA, USA) was used to measure the absorbance at 450 nm wavelength. These assays were performed in triplicate.

### Colony Growth Assay

Approximately 2,000 stably transfected cells were planted on 6-mm plates in each well and nurtured for 15 days. The media was replaced after every 3 days. Cells were washed with PBS twice before harvest and then subjected to hematoxylin staining. Cell colonies were counted using imagej software (Co. Bharti Airtel Ltd, Version. 1.52n) and colonies with > 100 cells were counted. Every assay was executed in triplicate.

### Cell Viability Assay

Cell viability was determined by the trypan blue exclusion assay. The cell suspension was mixed with trypan blue dye (Sigma, USA) and observed under the microscope within 3 minutes. Cell viability was determined by dividing the number of non-stained (viable) cells by the number of total cells counted. In addition, flow cytometry analysis were also used to test the cell viability. Cells were stained with Annexin-FITC and propidium iodide (PI) for 15 min (Biolegend, USA) according to the manufacturer’s protocol. Cells were placed in ice and analyzed using a Beckman Coulter Cytoflex.

### Cell Cycle Analysis

The stably transfected cells (1*10^6^) were seeded at 6-well plates until 60%–70% confluence with the culture medium. The cells were then collected *via* trypsinization, suspended again in PBS, and immobilized overnight at 4°C in 70% cold alcohol. Before cell cycle analysis, bovine pancreatic RNase (2 μg/ml; Sigma) was added to the cells at 37°C for 30 min, and the cells were then incubated in propidium iodide (20 μg/ml; Sigma) for 20 min. BD LSRII Flow Cytometry System and FACSDiva software (BD Bioscience, Franklin Lakes, USA) were used for analysis. NovoExpress software package was also used to assess the information, and the cell cycle distribution was presented as percentage of cells in G0/G1, S, and G2/M populations. These assays were repeated in triplicate.

### Tumor Xenograft Experiments

Four-week-old male NOD/SCID nude mice were acquired from Hunan SJA Laboratory Animal Co., Ltd., Changsha, Hunan, China. The animals were kept in a pathogen-free environment at The Institutional Animal Care and Use Committee of Affiliated Cancer Hospital, Xiangya School of Medicine, Central South University. Stable transfected T24-ADNP-knockdown/T24-ADNP-negative control and TCCSUP-ADNP-overexpression/TCCSUP-ADNP-vector was hypodermically introduced (1×10^6^ cells per injected) in the right axillary fossa of the NOD/SCID nude mice respectively (5 mice in each group). The tumor length (L) and width (W) were measured using a caliper, and then the individual volume was computed as per (L×W2)/2 equation. The mice injected with T24 were sacrificed on the 28th day and the others on the 42nd days, and the tumors were resected, measured, segmented, and then stained with anti-ADNP antibody, anti-Ki67, anti-cyclin D1, anti-CDK4, and anti-CDK6. Concurrently, the expression of ADNP, Ki-67, cyclin D1, CDK4, and CDK6 in isolated tissue were detected.

### Statistical Analysis

The obtained data were resultant of at least 3 separate assessments and were presented as mean ± S.D. The significance of immunostaining scores in human paraffin-fixed tissues were tested using one-way ANOVA followed post hoc Bonferroni test. Two-way ANOVA tests were used to identify the significance of cell proliferation curve and mice tumorigenesis curve among the various groups, and the post hoc Bonferroni test was used for pairwise comparisons. The group comparisons of mRNA and protein expression, cell colonies, cell cytometry, the final weight and volume of mice tumor, and the immunostaining scores of mice tumor were performed using Student’s t test. The weighted Fleiss-Cohen (quadratic) kappa statistics were used to assess the inter-observer agreement (24). The correlation between ADNP expression and clinicopathological features was examined *via* Chi-squared test. Survival arcs were drawn according to Kaplan-Meier analysis and evaluated using log-rank test. Survival data were analyzed using univariate and multivariate Cox regression analysis. All statistical analyses were performed using SPSS 21.0 (SPSS Incorporated, Chicago) statistical software package, and P < 0.05 was considered statistically significant.

## Result

### High ADNP Expression in Human BC

**Figure 1:**
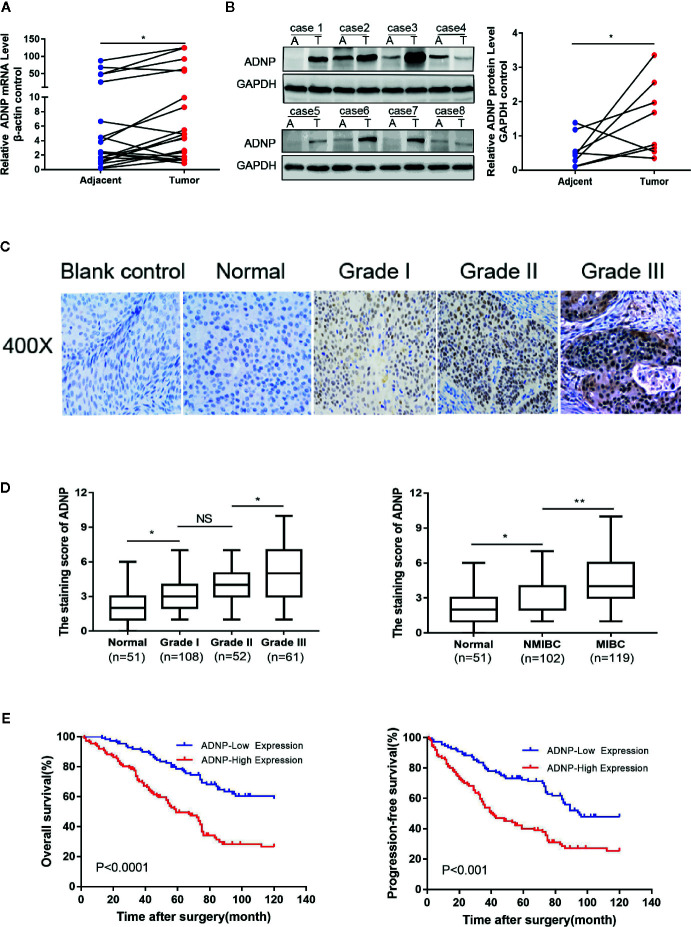
ADNP is upregulated in bladder cancer. **(A)** Real-time PCR analysis of ADNP mRNA expression in 20 paired bladder tumor tissues (Tumor) and their adjacent normal tissues (Adjacent). The average ADNP mRNA expression was normalized to the expression of β-actin. **(B)** ADNP protein expression in 8 paired bladder tumor tissues (T) and their adjacent normal tissues (A) determined *via* western blot analysis. GAPDH was detected as a loading control in the western blot analysis. **(C)** Representative images of ADNP expression in normal bladder tissues and different grades of bladder cancer (scale bar: 50 μm; magnification: ×400). **(D)** Quantification of immunohistochemical analysis of ADNP expression in 51 normal bladder mucosa and in 108 Grade I, 52 Grade II, and 61 Grade III bladder cancer tissues (left). Quantification of the average staining score for ADNP in 51 normal bladder mucosa and in different clinical stages of bladder cancer (102 NMBC, 119 MIBC) (right). **(E)** Kaplan-Meier curves of overall survival (left) and progression free survival (right) for the 221 patients with bladder cancer stratified by high and low expression of ADNP. **P < 0.01, *P < 0.05. NS, no significance.

To detect ADNP expression in BC tissue samples, we analyzed BC tissue pairing (T) and the pair of adjacent normal BC tissue (A) *via* RT-PCR. Compared with normal bladder urothelial tissue, the ADNP mRNA expression of BC tissues tended to be upregulated (P < 0.05, **Figure 1A**). Similarly, western blot analysis revealed that ADNP protein expression in human primary bladder tumor was also significantly upregulated (**Figure 1B**). These findings indicate that ADNP is highly expressed in BC.

### Upregulated ADNP Expression Is Associated With Advanced Clinicopathological Characteristics of BC

We examined the relationship between ADNP and the clinicopathological characteristics of bladder urothelial carcinoma. The weighted Fleiss-Cohen (quadratic) kappa value was 0.893. **Table 1** describes the medical data of 221 paraffin-embedded BC specimens. ADNP expression was significantly associated with pathological level (P = 0.009), T stage (P < 0.001), N stage (P= 0.007), and mortality status (P < 0.001) (**Table 1**), but not with age (P = 0.832), sex (P = 0.214), multifocal growth (P = 0.196), and tumor size (P = 0.508). Furthermore, immunohistochemical quantitative results indicated that ADNP expression was higher in the 221 cases of BC than in the 51 samples of normal bladder tissues (**Figures 1C,D**). Further, ADNP expression was higher in advanced clinical stage (MIBC) and pathological grade (Level III) BC than the early clinical stage (NMBC) and low pathological grade (Levels I and II) BC (**Figure 1D**). These results show that high ADNP expression is significantly associated with advanced clinicopathological features.

**Table 1:** ADNP expression in bladder cancer and its correlation with clinicopathological characteristics.

Characteristics ADNP	n (%)	ADNP		Pearson correlation	*P-*value
		Low (n = 110)	High (n = 111)		
**Age (y)**				0.014	0.832
≥ 60	96 (43.4%)	47	62		
< 60	125 (56.6%)	63	49		
**Sex**				-0.084	0.214
Male	180 (81.4%)	86	94		
Female	41 (18.6%)	24	17		
**pathological grade**				0.199	0.009
1	108 (48.9%)	65	43		
2	52 (23.5%)	22	30		
3	61 (27.6%)	23	38		
**T stage**				0.363	<0.001
Ta	49 (22.2%)	46	2		
T1	53 (24.0%)	20	33		
T2	88 (39.8%)	34	54		
T3	16 (7.2%)	5	11		
T4	15 (6.8%)	5	10		
**N stage**				0.182	0.007
Negative	179 (81.0%)	97	82		
Positive	42 (19.0%)	13	29		
**Tumor multiplicity**				-0.087	0.196
Unifocal	134 (60.6%)	62	72		
Multifocal	87 (39.4%)	48	39		
**Tumor size(cm)**				0.045	0.508
≥ 3	75 (33.9%)	75	71		
< 3	146 (66.1%)	35	40		
**Mortality status**				0.285	<0.001
Alive	77 (34.8%)	53	24		
Dead	102 (46.2%)	37	65		
Lost to follow-up	32 (14.5%)	10	22		

### High ADNP Expression Is Associated With Poor Outcome

Immunohistochemistry was used to detect ADNP expression in 221 samples of paraffin-embedded tissues to determine the frequency of ADNP overexpression in BC tissues. In the 221 samples, 108 were pathological grade I; 52, pathological grade II; and 61, pathological grade III (**Table 1**). We found there was higher ADNP expression in BC specimens when compared with normal bladder urothelial tissues. Quantitative analysis showed that the expression of ADNP was highly upregulated in the 221 BC specimens. In Cox regression analysis, we evaluated the potential of ADNP as a predictive factor of prognosis; it showed high ADNP expression significantly increased the risk of mortality in both univariate (P < 0.001; **Table 2**) and multivariate analyses (P = 0.001). The Kaplan-Meier survival curve showed that the overall survival of patients with high ADNP expression was lower than that of patients with low ADNP expression, and the survival without disease progression show the same (**Figure 1E**, P < 0.001). These results support that upregulated ADNP expression is related to poor prognosis in BC.

**Table 2:** Univariate and multivariate Cox regression analyses of prognostic factors in bladder cancer.

Variables	Univariate analysis			Multivariate analysis		
	HR	95% CI	*p*-Value	HR	95% CI	*p*-Value
**Age**	0.863	0.591–1.256	0.863	–	–	–
**Sex**	0.862	0.499–1.368	0.458	–	–	–
**Pathological grade**	1.833	1.461–2.299	<0.001	1.364	1.059–1.759	0.016
**T stage**	1.980	1.668–2.350	<0.001	1.882	1.321–2.680	<0.001
**N stage**	1.774	1.147–2.744	0.010	1.899	1.223–2.948	0.004
**Tumor multiplicity**	0.956	0.653–1.399	0.817	–	–	–
**Tumor size**	1.970	1.354–2.864	<0.001	2.113	1.427–3.129	<0.001
**ADNP**	2.627	1.779–3.879	<0.001	1.944	1.330–2.990	0.001

HR, hazard ratio; CI, confidence interval.

### ADNP Regulates Bladder Cancer Cell Proliferation

**Figure 2:**
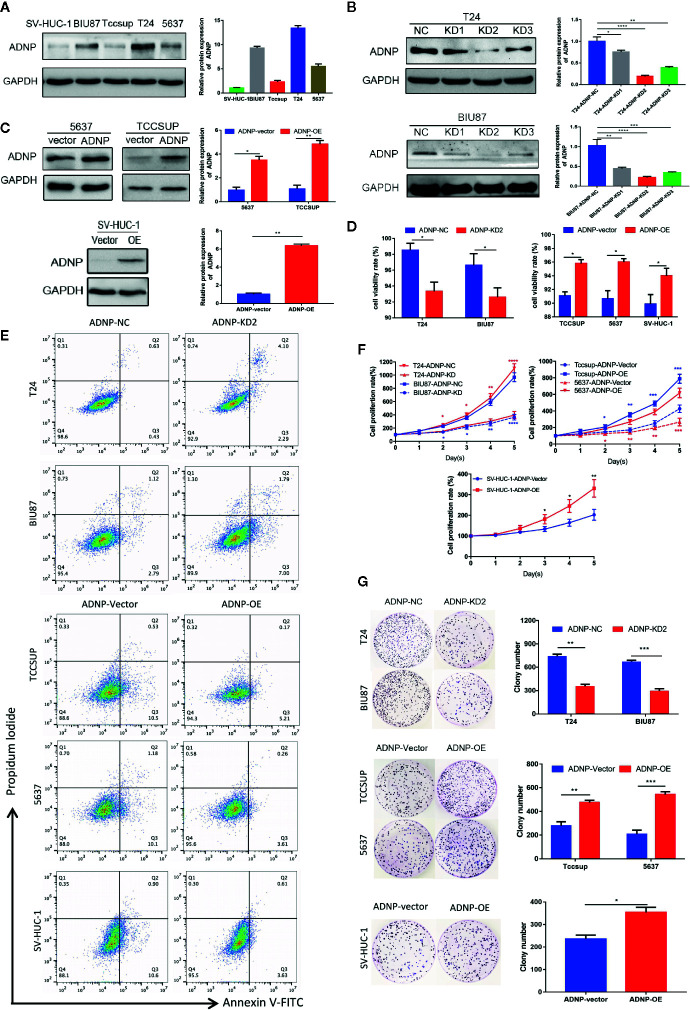
ADNP modulates proliferation of bladder cancer cells. **(A)** Western blot analysis of ADNP basic expression in 4 different bladder cancer cell lines (T24, BIU87, TCCSUP, 5637) and 1 normal urothelial cell line (SV-HUC-1). **(B)** The efficiency of ADNP knockdown in T24 and BIU87 cells were determined by Western blot **(C)** The efficiency of ADNP overexpression in 5637, TCCSUP, and SV-HUC-1 cells were determined by Western blot. **(D)** Cell viability of bladder cells and SV-HUC-1 cells were determined by the trypan blue exclusion assay. **(E)** Representative images of flow cytometric analysis were used to show viability rates after ADNP knockdown or overexpression. **(F)** CCK8 assays revealed the proliferation of indicated bladder cancer cells. **(G)** Colony formation assay test for tumorigenesis in indicated bladder cancer cells. The results are presented as the mean ± SD. ****P < 0.0001, ***P < 0.001, **P < 0.01, *P < 0.05.

In western blot analysis to detect ADNP protein expression in BC cell lines and normal urothelial cell line, ADNP was significantly increased in T24 and BIU87 cells and had relatively low expression in 5637, TCCSUP, and SV-HUC-1 cells (**Figure 2A**). Therefore, we constructed ADNP-knockdown cells with three specific sites by lentivirus, verified the knockdown efficiency with western blot analysis, and selected the lowest-knockdown-degree cell for a series of functional experiments (**Figure 2B**). In addition, we overexpressed ADNP in 5637, TCCSUP, and SV-HUC-1 cells (**Figure 2C**). Trypan blue exclusion test and flow cytometry showed the downregulation of ADNP significantly decreased the cell viability in T24 and BIU87 cells, while overexpressed ADNP markedly increased the proportion of viable cells in TCCSUP, 5637, and SV-HUC-1 cells (**Figures 2D, E**). CCK8 assay showed that ADNP knockdown significantly slowed down the growth speed of T24 and BIU87, while overexpressed ADNP significantly increased the growth rate of BC cells and normal urothelial cells (**Figure 2F**). Further, compared with blank transfected cells, the downregulation of ADNP markedly reduced the average quantity of colonies in the colony growth experiment, while the overexpression of ADNP increased the clone number in TCCSUP, 5637, and SV-HUC-1 cells (**Figure 2G**). These findings show that ADNP can stimulate proliferation of BC cells.

### ADNP Accelerated G1-to-S Cell Cycle Transformation 

**Figure 3:**
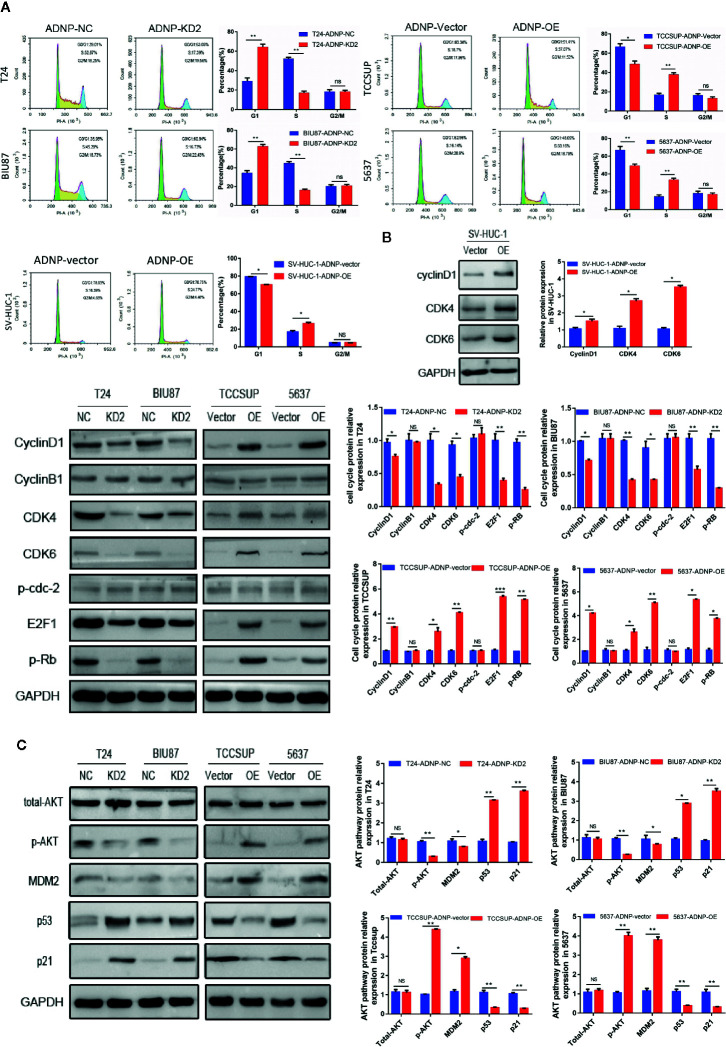
ADNP is involved in transition from G1 phase to S phase in the cell cycle and regulates proliferation of bladder cancer cells through the activation of the AKT-MDM2-p53 signaling pathway. **(A)** Representative images of flow cytometry analysis in indicated bladder cancer cell lines, and quantification of cell cycle analysis in indicated bladder cancer cell lines. **(B)** Western blot analysis of cyclin B1, cyclin D1, CDK4, CDK6, p-cdc-2, E2F1, and p-Rb expression in indicated cells. **(C)** Western blot analysis of phosphorylated Akt (p-Akt), total Akt, MDM2, p53, and p21 protein in the indicated bladder cancer cell lines. ***P < 0.001, **P < 0.01, *P < 0.05. NS, no significance.

To further explore the role of ADNP in stimulating cell propagation, flow cytometry was applied in cell analysis. ADNP knockdown markedly lowered the percentage of G0/G1 peak cells and improved the percentage of S peak cells (**Figure 3A**). However, there was no significant difference in G2/M phase ratio, and ADNP overexpression showed the opposite result, suggesting that ADNP can accelerate the G1 to S phase conversion of BC and normal urothelial cells (**Figure 3A**). In addition, western blot analysis showed that the expression of Cyclin D1, CDK4, CDK6, p-RB, and E2F1 in ADNP-overexpressed bladder cell lines was upregulated, while the expression of them was downregulated in ADNP-knockdown cells. Overexpressing ADNP also led to the significant increase of CyclinD1, CDK4, and CDK6 in SV-HUC-1 cells. In addition, changing ADNP expression did not have a significant impact on Cyclin B1 and p-cdc-2 expression (**Figure 3B**), both of which were promoters of G2-to-M transformation, suggesting that ADNP further increased BC cell propagation by accelerating the G1-S phase transformation.

### ADNP Activates AKT-MDM2-p53 Signaling Pathways

To determine the molecular dynamics regulating the cell cycle, several key proteins of classical signaling pathways were detected. After altering ADNP expression, p-AKT expression was higher in T24 and BIU87 cells, but lower in 5637 and in TCCSUP cells. Meanwhile, there were no significant changes in total AKT expression (**Figure 3C**). In addition, the activation of downstream target protein MDM2 was correlated with the degree of AKT phosphorylation, and the upregulation of MDM2 mediated the decrease of p53 and its downstream protein p21. These results suggest that ADNP may regulate p21-induced cell cycle transformation by activating the AKT/MDM2/p53 pathway, thereby promoting the propagation of BC cells.

### ADNP Regulates Bladder Cancer Tumorigenesis In Vivo

**Figure 4:**
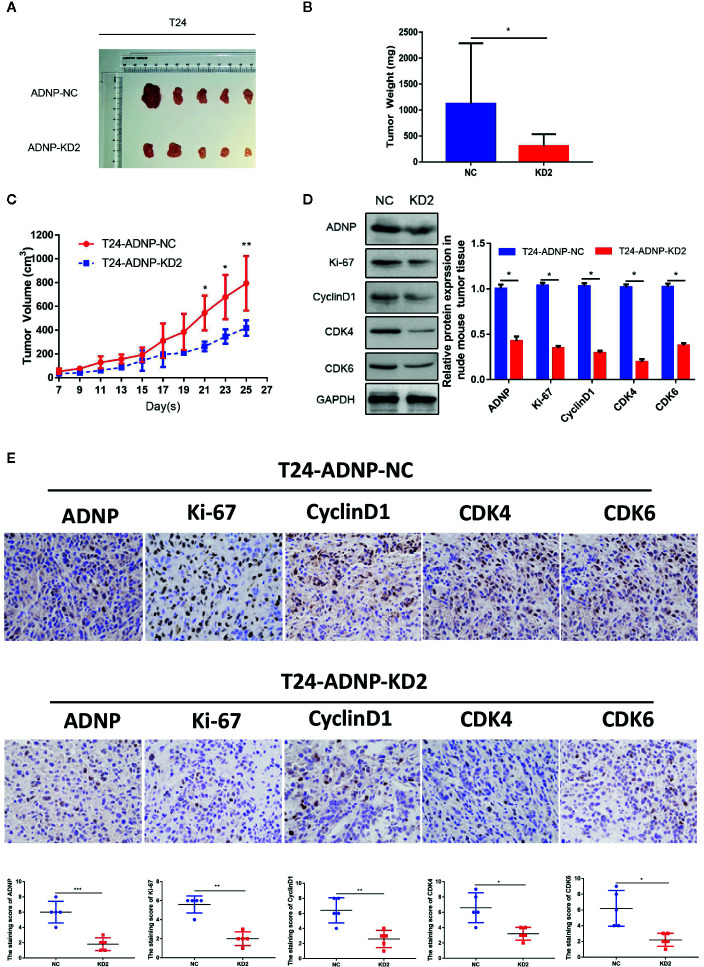
ADNP modulates growth of bladder cancer in vivo. **(A)** Images of excised tumors from five NOD/SCID mice at 25 days after injection with non-silencing shRNA cells (NC) and ADNP-sh2-transfected cells (KD2). **(B)** Average weight of excised tumors. **(C)** Tumor volumes were measured every 2 days. **(D)** Western blot analysis of ADNP, Ki-67, Cyclin D1, CDK4, and CDK6 expression in excised xenograft tumors. **(E)** Representative images of sections sliced from indicated tumors and stained with anti-ADNP, anti-Ki67, anti-Cyclin D1, anti-CDK4, and anti-CDK6. **P < 0.01, *P < 0.05.

To verify the outcome of tumorigenesis experiments in vitro, xenograft bladder tumor models were constructed in NOD/SCID nude mice using T24.The final volume, weight, and tumor growth curve of the transplanted tumor (**Figures 4A–C**) revealed that compared with the negative control group, T24 cells with ADNP knockdown had significantly lower capability of tumor formation. In addition, western blot analysis confirmed that the expression of ADNP, Ki-67, Cyclin D1, CDK4, and CDK6 in T24 BC cells with ADNP knockdown was considerably lower than those in the negative control (**Figure 4D**). Similarly, immunohistochemical results showed that ADNP knockdown group had lower expression of ADNP, Ki-67, CyclinD1, CDK4, and CDK6 compared with the control group (**Figure 4E**). These in vivo results indicate that ADNP might play a key role in BC tumorigenesis.

### Effect of ADNP on Proliferation and Cell Cycle Could Be Rescued by MK-2206 2HCI

**Figure 5:**
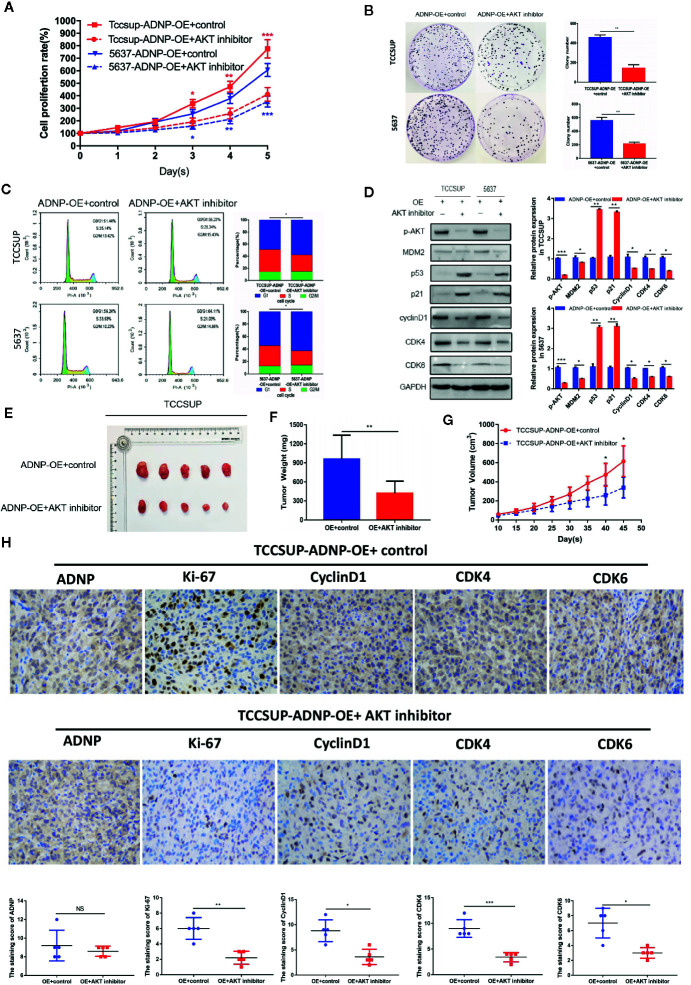
Effect of ADNP on proliferation and cell cycle could be rescued by MK-2206 2HCI. **(A)** TCCSUP cells with ADNP overexpression were treated with the AKT inhibitor MK-2206 for 24 h, and CCK8 assays revealed the role of AKT in the proliferation of ADNP-transduced cells. **(B)** Colony formation assays revealed the role of AKT in the tumorigenesis of ADNP-transduced cells. **(C)** Flow cytometric assays revealed the role of AKT in the G1-to-S transition of ADNP-transduced cells. **(D)** Western blot analysis revealed the role of AKT in the downstream cell cycle-associated genes of ADNP-transduced cells. **(E)** Images of excised tumors from five NOD/SCID mice at 45 days after being handled with MK-2206 2HCI and PBS (control). **(F)** Average weight of excised tumors. **(G)** Tumor volumes were measured every 5 days. **(H)** Representative images of sections sliced from indicated tumors and stained with anti-ADNP, anti-Ki67, anti-cyclin D1, anti-CDK4, and anti-CDK6. ***P < 0.001, **P < 0.01, *P < 0.05. NS, no significance.

To elucidate the role of AKT in ADNP-induced cycle acceleration and proliferation of BC cells, the AKT-specific inhibitor MK-2206 2HCI was used to treat 5637 and TCCSUP BC cells that overexpressed ADNP. CCK8 experiment and clone growth experiment results show that the AKT inhibitor can inhibit the proliferation in BC cells (**Figures 5A, B**). Moreover, compared with the blank group, the percentage of S phase cells was significantly lower in the AKT inhibitor treatment group (**Figure 5C**). Further, we found that MK-2206 2HCI can reverse the effect of ADNP on p-AKT, MDM2, p53, p21, cyclinD1, and CDK4 and CDK6 (**Figure 5D**). In vivo experiment, the final volume, weight and tumor growth curve of the transplanted tumor (**Figures 5E–G**) showed that TCCSUP cells treated with MK-2206 2HCI had significantly lower capability of tumor formation compared with the control group. Moreover, immunohistochemical results confirmed that the expression of Ki-67, Cyclin D1, CDK4, and CDK6 in the TCCSUP cells handled with MK-2206 2HCI was considerably lower than those in the control; there was no significant difference on the expression of ADNP between the two groups (**Figure 5H**). Collectively, these results suggest that the changes in cell cycle and proliferation mediated by ADNP expression are related to the AKT-MDM2-p53 signaling pathway in BC cells.

## Discussion

Here, we found that ADNP expression was upregulated in BC, and significantly correlated with high pathological grade and advanced clinical stage. ADNP stimulates the AKT-MDM2-p53 pathway and enhances binding of cyclin D1 to CDK4 or CDK6, accelerating the cell cycle transition from G1 phase to S phase. Furthermore, we demonstrate that ADNP can promote the growth of bladder cancer in vivo.

The significance of ADNP expression in BC is unclear. Although the copy number of the ADNP gene has been reported to be amplified in BC (13), no study has explored the significance of ADNP mRNA and protein expression in the clinicopathology of BC. In this study, we found that the mRNA and protein expression of ADNP were upregulated compared with normal bladder urothelial tissues, and overexpression of ADNP protein was significantly associated with higher pathological grade, advanced clinical stage, and poor prognosis in BC patients. Consistent with our observations, DNA amplification and overexpression of the transcription factor ADNP were identified in high-grade and poorly prognostic ovarian cancer (25).

ADNP encodes a protein involved in embryonic development and brain formation (9). In previous studies, ADNP was associated with the de novo mutation that leads to autism-like spectrum disorder (18). However, a recent study suggested that negative ADNP expression in the colon activates the WNT signaling pathway, promoting colon cancer cell migration, invasion, and proliferation (15). We found that ADNP expression was significantly correlated with high pathological grade and advanced clinical stage, indicating that ADNP may be an oncogene of BC that can lead to tumor cell proliferation and tumorigenesis.

To test our hypothesis, we studied ADNP function in BC in vivo and in vitro. We found the evidence in our study that ADNP can significantly promote tumor cell growth and proliferation. The infinite proliferation in cells is an important factor leading to the development of tumors. Alessandro et al. (17) showed that the activation of the ADNP signaling pathway increases the survival ability of malignant glioma cells in harsh environments and participates in the resistance to cell death, which is consistent with our experimental results.

In general, infinite growth is caused by the increase of cell viability or the disruption of cell cycle checkpoints (26). To investigate whether this effect is related to cell viability, we performed trypan blue exclusion tests and flow cytometry, which showed ADNP lead to increased cell viability. In line with our study, ADNP was also proved to increase the cell viability to resist the toxicity induced by H_2_O_2_ in malignant peripheral nerve sheath tumor (17). The disorder of cell cycle is the initial cause of abnormal growth of tumor cells and the important step of obtaining infinite proliferation ability of tumor (26). After the cell cycle is disturbed, cell division is no longer regulated by G1/S and G2/M checkpoints, and cells can obtain stronger proliferation capacity (27, 28). A study recently showed that the mice with ADNP haplo-insufficiency exhibit the decrease ability of wound healing due to the broken cell cycle progression, and the reactivation of ADNP in mouse models increased the ability of wound healing and accelerated cell cycle progression (29). Moreover, ADNP was identified to be an oncogenic mediator of cell proliferation through dysregulation of cell cycle checkpoints in high-grade serous ovarian cancer (25). In our study, flow cytometry showed that ADNP significantly accelerated transition of BC cells from G1 phase to S phase. Meanwhile, our Western blot analysis indicated that ADNP significantly increased the expression of Cyclin D1, CDK4, CDK6, phosphorylating retinoblastoma (p-Rb), and E2F1. Cyclin D1 is mainly expressed in the G1 phase of the cell cycle, and it can combine with cell cycle protein CDK4 or CDK6 to form active cell cycle protein complexes, such as p-Rb protein and E2F, which crosses the cell cycle checkpoint of the G1 phase to S phase (30, 31).

In many types of tumor cells, AKT can be activated by growth factor receptors, tenin homologous proteins, and mutations in tumor suppressor phosphatase signals, transmitting effective proliferation signals (32). AKT can promote tumor cell proliferation by accelerating the progress of cell cycle G1-S by isolating p21 in the cytoplasm and stabilization of cyclin D1 (33, 34). Previous studies show p-AKT can directly promote the degradation of p53 protein by nuclear MDM2 and downregulate the activity of p53, resulting in an imbalanced microenvironment that promotes cancer cell proliferation but inhibits apoptosis (35, 36). Our study shows that ADNP activated the AKT-MDM2-p53 signaling pathway, mediated the degradation of p21 and the stability of cyclin D1, and promoted the proliferation of tumor cells. Consistent with our research, a number of studies have reported that ADNP plays an important role in regulating cell growth, proliferation in some types of sarcomas and neuronal tissue as well as modulating AKT signaling pathway (25). AKT signaling also plays an important role in CD8^+^T cell differentiation and regulatory T cell survival (37–39). Loss or weakening of AKT signaling would result in transcriptional rearrangement of differentiated cytotoxic T lymphocytes, transforming effector cells into memory cells to enhance the anti-tumor effects in the immune system (40, 41). In a xenograft study, MK-2206 2HCI treatment in mice with ovarian cancer cell lines resulted in 60% growth inhibition (42). Meanwhile, the addition of AKT inhibitor blocked the radiation-induced Treg cell survival in bladder cancer cell, which can inhibit CD8^+^ T cells in the tumor tissue to compromise the antitumor activities (43). We found that the effect of ADNP on tumor proliferation was rescued by AKT inhibitors, suggesting that ADNP promotes cell proliferation mainly through activation of the AKT pathway in BC. Previous studies have reported that ADNP can activate the AKT pathway to promote the neuronal growth and differentiation, which is related to the up-regulation of the expression of AKT phosphorylated kinase (19), and this mechanism may also exist in bladder cancer. Nevertheless, further studies are warranted to replicate and extend these findings.

In conclusion, the mRNA and protein expressions of ADNP were up-regulated in human bladder cancer and reveal its role as a tumor promoter with effects on tumor cell proliferation as well as cell cycle with the activation of AKT pathway. Accordingly, these findings provide the evidences that ADNP expression can be an independent predictor of poor prognosis in patients with BC, and a candidate therapeutic target for novel molecular therapy.

## Data Availability Statement

All datasets generated for this study are included in the article/supplementary material.

## Ethics Statement

The studies involving human participants were reviewed and approved by The study involving human participants was approved by the ethics committee of the Affiliated Cancer Hospital of Xiangya School of Medicine, Central South University. Patients/participants provided written informed consent for their participation in this study. The patients/participants provided their written informed consent to participate in this study. The animal study was reviewed and approved by The animal research was examined and approved by the animal ethics committee of the Affiliated Cancer Hospital of Xiangya School of Medicine, Central South University.

## Author Contributions

YX and SZ designed the research. YL and QD collected the data. GF and JC interpreted the data. SZ, ZX, and YW carried out the experiment. SZ drafted the manuscript. YX, YZ, and WH critically revised the manuscript. All authors contributed to the article and approved the submitted version.

## Funding

This study was funded by the Key Research and Development Projects of Hunan Province (grant number 2018SK2125 and 2018SK2120) and the Key Research Project of National Cancer Center (grant number NCC201818A55).

## Conflict of Interest

The authors declare that the research was conducted in the absence of any commercial or financial relationships that could be construed as a potential conflict of interest.
